# Safety, Immunogenicity, and Efficacy of Cytomegalovirus Vaccines: A Systematic Review of Randomized Controlled Trials

**DOI:** 10.3390/vaccines13010085

**Published:** 2025-01-17

**Authors:** Manuela Chiavarini, Anita Genga, Giorgia Maria Ricciotti, Marcello Mario D’Errico, Pamela Barbadoro

**Affiliations:** 1 Department of Health Sciences, University of Florence, Viale GB Morgagni 48, 50134 Florence, Italy; manuela.chiavarini@unifi.it; 2 Department of Biomedical Sciences and Public Health, Section of Hygiene, Preventive Medicine and Public Health, Polytechnic University of the Marche Region, 60126 Ancona, Italy; m.m.derrico@staff.univpm.it (M.M.D.); p.barbadoro@staff.univpm.it (P.B.)

**Keywords:** systematic review, cytomegalovirus, vaccine, congenital infection, latency, glycoproteins, pregnancy, clinical trials, immunogenicity, efficacy

## Abstract

**Background/Objectives:** Cytomegalovirus (CMV) is widespread and mostly causes asymptomatic infections in immunocompetent hosts, but it may lead to severe and life-threatening diseases in immunocompromised individuals, such as transplant patients and congenitally infected children, representing a significant public health concern. Although there are no licensed CMV vaccines, the development of a CMV vaccine is considered a high priority due to its potential to reduce the burden associated with CMV-related complications, and several approaches are under investigation. The objective of this systematic review was to synthesize the evidence on various CMV vaccines currently under clinical development. **Methods**: According to the PRISMA guidelines (PROSPERO ID: CRD42024516601), a comprehensive literature search was conducted to identify all the randomized controlled trials that have evaluated the safety, immunogenicity, and efficacy of vaccine candidates compared to a placebo. A total of 26 studies were identified: 11 on transplant patients and 15 on healthy individuals. **Results**: Several vaccine candidates have shown encouraging results in terms of safety and specific immune responses, notably adjuvanted gB vaccines and DNA vaccines targeting gB and pp65. The results were divided into RCTs on healthy individuals and those on transplant recipients, because the CMV-specific immune response to a vaccine is complex and varies depending not only on the type of vaccine, but also on the immunological status of the individual. **Conclusions**: Challenges remain in achieving broad efficacy across diverse populations, particularly for immunocompromised patients. Thus, the present work seeks to support future decisions and guide further research in the development of an effective and widely available CMV vaccine.

## 1. Introduction

Cytomegalovirus (CMV) is a pervasive herpesvirus that is highly prevalent worldwide. A recent systematic review estimated a CMV global seroprevalence of 83% in the general population, from approximately 60% in developed countries to more than 90% in many developing countries [1].

In healthy individuals, CMV remains latent, and the immune system controls the virus without any significant long-term health problems, except in special circumstances like pregnancy [2]. CMV seroprevalence is observed in approximately 86% of women of childbearing age [1].

CMV-associated morbidity and mortality predominantly impact two groups: congenitally infected newborns and immunosuppressed individuals. In fact, CMV can cross the placental barrier and cause congenital disease in the developing fetus, which may result in the lasting impairment of cognitive development and is the primary cause of non-genetic sensorineural hearing loss [3].

In immunocompromised individuals, such as solid organ transplant (SOT) or hematopoietic stem cell transplant (HSCT) recipients, CMV can become a serious problem, as a CMV infection is one of the most frequent infectious complications after an allogeneic HSCT, and it can cause end organ disease [4,5].

Roughly half of transplant recipients release CMV through bodily fluids such as saliva and urine at some point after receiving an organ transplant. This typically starts within the first month post-surgery. The highest levels of viral excretion occur during the second and third months after transplantation and may coincide with the onset of the disease. The immunosuppressive drugs used to prevent transplant rejection lower the body’s ability to control the virus, leading to the reactivation of latent CMV or new infections from the donor organ. Given the severe health complications associated with CMV infections in immunocompromised individuals, organ transplant recipients, and congenitally infected newborns, CMV represents a significant health burden [6].

Clinically, there are a number of antiviral medications used to treat acute CMV disease. However, during pregnancy and under conditions of immune suppression, antivirals are not the standard of care [7].

With humans as the sole reservoir of infection, prevention efforts are critical. Thus, the development of a CMV vaccine could have significant public health benefits by reducing the incidence of CMV-related complications, improving the quality of life for affected populations, and lowering healthcare costs associated with managing CMV infections and their long-term effects. The Institute of Medicine (IOM) has consistently rated the development of a CMV vaccine as a high priority due to its potential to prevent congenital infections and reduce healthcare burdens associated with CMV-related complications [8].

Currently, several vaccine candidates are in development, with the aim of preventing both initial CMV infections and the reactivation of the virus in individuals who are already carriers. These vaccines employ various strategies to stimulate a robust immune response capable of neutralizing the virus or controlling its re-emergence in the host. Multiple approaches are under investigation to enhance immunity against CMV, such as DNA vaccines, vectored vaccines, live attenuated vaccines, recombinant subunit vaccines, peptide vaccines, and mRNA vaccines [8].

The objective of this work was to provide a comprehensive synthesis of the current evidence concerning CMV vaccines under development, focusing on their safety profiles, immunogenicity, and efficacy. This analysis aimed to consolidate the findings from ongoing research to offer insights into how these vaccines perform across various clinical parameters, ultimately contributing to a better understanding of their potential role in preventing CMV infections. By evaluating the strengths and limitations of each vaccine candidate, this work seeks to support future clinical decisions and guide further research in the development of effective CMV vaccination strategies.

## 2. Materials and Methods

### 2.1. Protocol Registration

The Preferred Reporting Items for Systematic Review and Meta-Analyses 2020 (PRISMA 2020) guidelines [9] were followed in conducting the systematic review, and it was registered in the International Prospective Register of Systematic Reviews (www.crd.york.ac.uk/PROSPERO/ (accessed on 4 March 2024), registration No: CRD42024516601).

### 2.2. Data Source and Search Approach

A thorough search of the literature was conducted from the creation of the databases to 31 January 2024. All original publications assessing the safety, immunogenicity, or efficacy of any CMV vaccine were found using the Medline/PubMed (http://www.ncbi.nlm.nih.gov/pubmed/), Web of Science (http://wokinfo.com/), Scopus (https://www.scopus.com/), and Cochrane Central Register of Controlled Trials (https://www.cochranelibrary.com/) databases. The Supplementary Material (Supplementary Table S1) reports the medical topic headings (MeSH) and key words used in the literature search. Only human research was included in the search and all the studies were published in the English language.

### 2.3. Eligibility Criteria

The publications were vetted by two authors using titles and abstracts. Any instances of a study being published twice were noted and subsequently eliminated. The full texts of potentially eligible papers were gathered and evaluated for inclusion based on the inclusion and exclusion criteria.

Articles that satisfied the following requirements were accepted:

1. Compared any CMV vaccine to a placebo in transplant recipients or healthy individuals.
2. Compared two or more groups using a randomized controlled trial (RCT) design, one of which received a placebo as a control and the other any CMV vaccine as an intervention.
3. Released results on the immunogenicity (a humoral or cell-mediated reaction), safety (any possible adverse effects), and efficacy (laboratory-confirmed CMV) of a CMV vaccine.

The most recent publication was selected if the same study was reported in several publications. Reviews and meta-analyses were excluded. Any disagreement was resolved by discussion or in consultation with a third author.

### 2.4. Data Extraction and Quality Assessment

Two authors (A.G. and G.M.R.) independently performed a data extraction and quality assessment for each included study. The first author’s last name, the year of publication, the nation, the type of vaccine, the size and characteristics of the study population (age, gender, and CMV serostatus), the trial phase, the size of the experimental and placebo arms, and the research outcomes were all taken from each included study. The safety, immunogenicity, and efficacy data were taken from the populations according to the protocol.

Two authors (A.G. and G.M.R.) independently evaluated the study quality using the Cochrane risk-of-bias tool (RoB-Tool 2) [10], and discrepancies were resolved through discussion and consultation with a third author.

The studies were assessed for potential bias across five critical areas: the randomization process, adherence to the intended interventions, the completeness of the outcome data, the accuracy of the outcome measurement, and the selection of the reported results. Each domain was assigned a judgment of low risk, some concerns, or high risk. The overall risk of bias for a study was categorized as low if all the domains were judged to have a low risk, as having some concerns if at least one domain raised minor concerns without reaching a high risk level, and as high if any domain was deemed high risk or if concerns in multiple domains significantly diminished the confidence in the findings. No studies were excluded due to quality concerns.

## 3. Results

### 3.1. Study Selection

**Figure 1:**
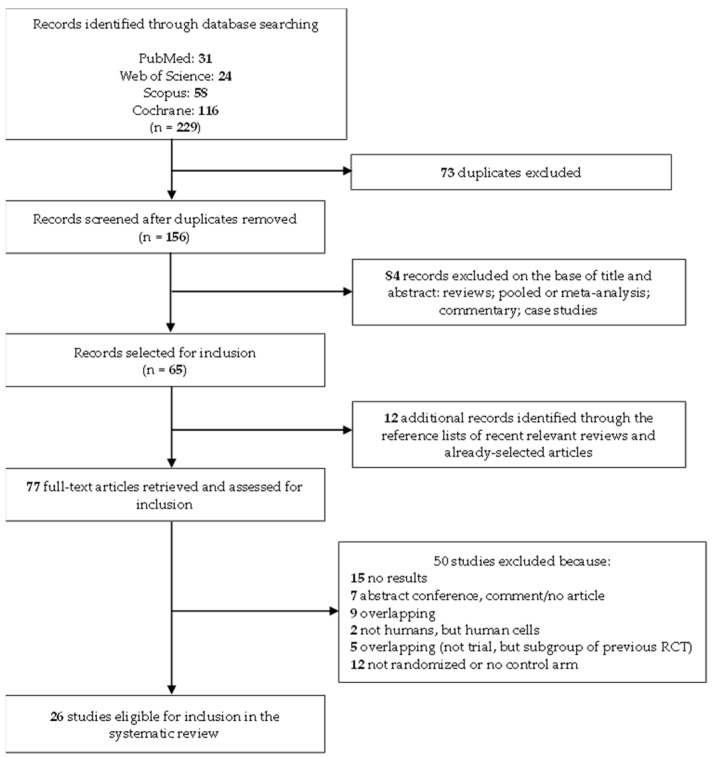
Flow diagram of the systematic literature search for RCTs on CMV vaccines.

The literature search identified 31 studies from PubMed, 24 from Web of Science, 58 from Scopus, and 116 from the Cochrane Library (Figure 1). After removing 73 duplicates, 156 unique records were screened based on their titles and abstracts. Of these, 84 articles were excluded for being reviews, pooled analyses, meta-analyses, commentaries, or case studies. This left 65 studies for a full-text evaluation. Additionally, 12 studies were identified through reference lists of selected articles and recent relevant reviews. Ultimately, 50 articles were excluded for not meeting the inclusion criteria: 15 lacked reported results, 7 were conference abstracts or comments, 9 presented overlapping results, 2 involved human cells rather than human subjects, 5 focused on subgroups of earlier RCTs, and 12 were not randomized or lacked a control arm. At the end of the selection process, 26 studies were eligible for inclusion in the systematic review [11,12,13,14,15,16,17,18,19,20,21,22,23,24,25,26,27,28,29,30,31,32,33,34,35,36].

### 3.2. Study Characteristics and Quality Assessment

The general characteristics of the 26 RCTs on CMV vaccines included in the systematic review are shown in Table 1a,b. A total of 11 studies involved transplant patients [11,12,13,14,15,16,17,18,19,20,21], and 15 studies involved healthy patients [22,23,24,25,26,27,28,29,30,31,32,33,34,35,36].

Table 1a summarizes the characteristics of the 11 studies involving transplant patients [11,12,13,14,15,16,17,18,19,20,21]. Among these, five studies were published between 1984 and 1994 [11,12,13,14,15], three between 2011 and 2018 [16,17,19], and three after 2020 [18,20,21].

All of the studies were conducted in the USA except one, which was conducted in the UK [19].

The types of vaccines were Towne live attenuated [11,12,13,14,15], DNA plasmid [16,17,18], recombinant subunit [19], vector vaccine [20] and peptide-based [21].

All the studies included an adult population, ranging from 18 to 75 years of age, except for two studies that included patients from 12 years of age [12,13]; the age was not reported in three studies [11,12,13,14,15].

The percentage of males ranged between 48% and 73%, and the gender of the included population was not reported in five studies [11,12,13,14,15].

The patients’ serostatus was both CMV-seropositive and CMV-seronegative in five studies [11,12,13,14,19], only seropositive in four studies [16,18,20,21], and only seronegative in two studies [15,17].

The patients were renal transplant candidates [11,12,13,14,15,17], renal or liver transplant candidates [19], or undergoing an HSCT [16,18,20,21].

There was one phase 3 study [18] and five phase 2 studies [16,17,19,20,21], while the phase of the study was not reported in five studies [11,12,13,14,15].

All the studies were randomized, were double-blinded, and compared an intervention arm, with the administration of the study vaccine, and a control arm, with the administration of a placebo.

The endpoints examined were the safety [11,13,14,16,17,18,19,20], immunogenicity [12,13,15,16,17,18,19,20,21], and efficacy [11,14,15,16,17,18,19,20,21].

Across the studies focused on this specific population, the vaccines demonstrated a favorable safety profile, with mild adverse effects reported overall and no significant differences between the vaccine and placebo groups [11,13,14,16,17,18,19,20].

Considering immunogenicity, five studies examined the T-cell responses to the vaccine versus the placebo [16,17,18,20,21]. Two studies found no significant difference between the groups [16,17], while one study showed a stronger T-cell response in the placebo group [18]. In contrast, the vaccine group showed a greater T-cell response in the remaining two studies [20,21]. Seven studies assessed the antibody titer responses [12,13,15,16,17,18,19]. One study indicated that the responses varied based on the donor and recipient serostatus [12]. Among seronegative recipients with seronegative donors, the titers substantially declined within one year post-immunization [12], while another study found antibody persistence for at least three years in the same donor–recipient combination [13]. In two studies, the antibody titer responses did not significantly differ between the vaccine and placebo arms [16,17], while two other studies observed a significant increase in the gB antibody titers [18,19].

Regarding efficacy, three studies reported a significantly lower severity of CMV disease among vaccinated patients [11,14,15], while one study found no difference in mild disease [15]. Another study did not observe a significant reduction in CMV end-organ disease and no improvement in the overall survival within one year [18]. One study reported a reduced risk of CMV events [20], though another found no significant difference [21]. Additionally, a significant reduction in the duration of CMV viremia was noted in one study [19], while another study observed a lower viremia rate [16], with no difference in the rates in a separate study [17]. In terms of antiviral therapy, a study showed a significant reduction in the need for CMV treatment [19], while there was no difference in another study [16].

**Figure 2:**
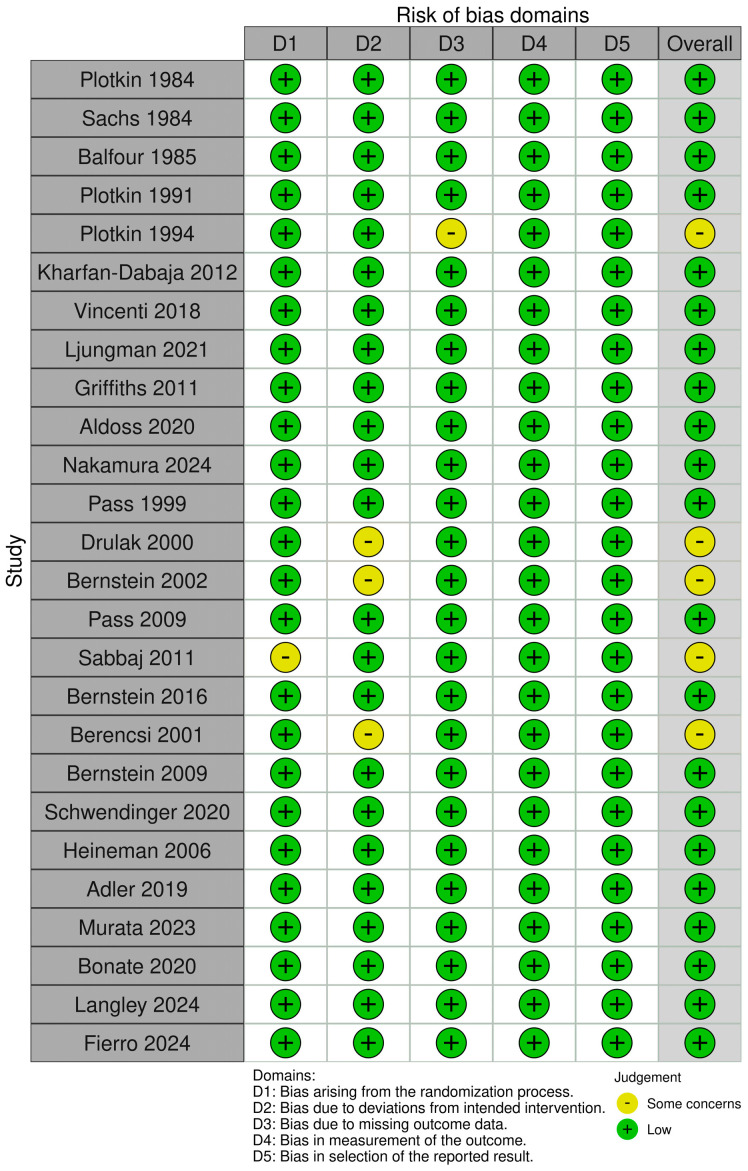
Risk of bias of included studies [11,12,13,14,15,16,17,18,19,20,21,22,23,24,25,26,27,28,29,30,31,32,33,34,35,36] (Cochrane risk-of-bias tool, RoB-Tool 2).

Ten included studies had a low risk of bias [90.9%], while one study raised some concerns due to missing outcome data [15] (Figure 2).

Table 1b summarizes the characteristics of the 15 studies involving healthy patients [22,23,24,25,26,27,28,29,30,31,32,33,34,35,36]. Among these, seven studies were published between 1999 and 2009 [22,23,24,25,28,29,31], three between 2011 and 2019 [26,27,32], and five after 2020 [30,33,34,35,36].

Most of the studies were conducted in the USA except two: one that was conducted in Japan [33] and one in Canada [35].

The types of vaccines were recombinant subunit [22,23,24,25,26,27], vector vaccine [24,28,29,30], Towne/Toledo live attenuated [31], V160 live attenuated [32,33], DNA plasmid [34], virus-like particles [35], and mRNA [36].

All the studies included an adult population ranging from 18 to 64 years of age, except for two studies that included patients from 14 years of age [25,26] and one that included adolescent patients from 12 to 18 years of age [27]. The age was not reported in one study [23].

In the studies involving both females and males, the percentage of males ranged between 18.5% and 72.2%; three studies involved only females [25,26,27] and one only males [33], while the gender of the population was not reported in two studies [23,24].

The patients’ serostatus was both CMV-seropositive and CVM-seronegative in five studies [28,32,33,34,36], only seronegative in seven studies [22,24,25,27,29,30,35], and only seropositive in three studies [23,26,31].

There were eleven phase 1 studies [22,26,28,29,30,31,32,33,34,35,36] and two phase 2 studies [25,27], while the phase of the study was not reported in two studies [23,24].

All the studies were randomized and compared an intervention arm, with the administration of the study vaccine, and a control arm, with administration of a placebo. Eleven studies were double-blinded [22,25,26,27,29,30,31,32,33,35,36], two were patient-blinded [23,34], and the blinding was not reported in two studies [24,28].

The endpoints examined were safety [22,23,24,25,27,28,29,30,31,32,33,34,35,36], immunogenicity [22,23,24,26,27,28,29,30,31,32,33,34,35,36], and efficacy [25,27,31].

Fourteen studies evaluated vaccine safety, consistently finding it well tolerated across all trials. Local and systemic reactions were generally similar between the vaccine and placebo groups [22,31], though two studies reported higher frequencies in the vaccine group [25,27]. Four studies noted increased pain at the injection site among vaccinated participants [22,31,32,34].

Regarding immunogenicity, eight studies examined the T-cell response, with seven showing a significant increase following vaccination [24,28,29,30,32,34,36], while one reported no change [31]. The humoral response was assessed in twelve studies, six of which reported notably higher levels of anti-gB antibodies in the vaccine group compared to the placebo group [22,23,24,26,27,35], with only one study observing no difference [34]. The neutralizing antibody levels were significantly elevated in the vaccine group in nine studies [22,24,29,30,32,35,36], although the increased levels were exclusive to CMV-negative [33] or CMV-positive [26] participants in two studies. One study found no significant change in neutralizing antibodies [31].

Two studies showed no difference in the efficacy between groups [27,31], whereas the infection rates per 100 person-years were reduced by 50% in the vaccine group in one study [25].

Eleven studies had a low risk of bias [73.3%], while four studies raised some concerns, three of which were due to deviations from the intended intervention [23,24,28] and one of which was due to the randomization process [26] (Figure 2).

## 4. Discussion

This systematic review examined 26 studies on the safety, immunogenicity, and efficacy of CMV vaccines. Of these, eight (30.7%) were published after 2020, which included two phase 2 studies [20,21] and one phase 3 study [18] involving transplant patients and five phase 1 studies on healthy individuals [30,33,34,35,36].

Phase 1 trials are the first stage in human population studies, and they aim to assess safety, tolerability, and preliminary data on immunogenicity; phase 2 trials establish the safety, optimal dosing, immunogenicity, and initial data on the efficacy in a larger population. Notably, phase 3 trials mark an important milestone by including a broader population sample, allowing safety, immunogenicity, and efficacy to be confirmed in a large-scale study.

The majority of the studies (88.5%) were conducted in the United States, highlighting a predominance of CMV vaccine research concentrated in a single country.

CMV vaccines aim to achieve different objectives based on the target population. For healthy individuals, the vaccine’s goal is to prevent an initial CMV infection and congenital CMV transmission. In contrast, for transplant recipients, the vaccine focuses on preventing CMV reactivation and reinfection following the transplantation.

Achieving a robust and comprehensive immune response, both humoral and cellular, is crucial for the vaccine’s effectiveness, as CMV-specific immune responses are complex and change depending not only on the type of vaccine, but also on the CMV serological status of the population.

CMV is known to evade several immune mechanisms for viral control and clearance through tissue-specific responses. Understanding the pathogenesis and the immune response to CMV infections, as well as the virus’s immune evasion strategies, is essential for developing an effective vaccine [37].

Despite several vaccine candidates and significant progress, no CMV vaccine has yet received regulatory approval, though the current research emphasizes two main vaccine types: DNA plasmid-based vaccines and protein-based vaccines.

DNA plasmid-based vaccines, which were used in two phase 2 trials [16,17] and one phase 3 trial [18] on transplant recipients, use plasmids—circular DNA molecules—as platforms to introduce one or more CMV antigens, eliciting antigen-specific immune responses. Within this category, the ASP0113 (previously known as VCL-CB01 or TransVax) vaccine, a bivalent hCMV DNA vaccine, stands out. This vaccine combines two plasmids encoding the pp65 and gB antigens, along with two adjuvants (poloxamer CRL1005 and benzalkonium chloride).

The results of a previous trial [16], with a significant reduction in the viral load endpoint (CMV viremia), provided the basis for a phase 3 trial in hCMV-seropositive recipients undergoing an allogeneic HSCT [18]. Although ASP0113 was generally well tolerated, with injection-site reactions being the most common side effect, it did not meet the primary or secondary endpoints. Specifically, it showed no statistically significant improvements in the overall survival or a reduction in CMV end-organ disease. However, a significant antibody response to the gB antigen was observed at the 12-month mark (*p* = 0.03), though no difference was found compared to the placebo overall (*p* = 0.112).

The second category involves protein-based vaccines, specifically the gB/MF59 vaccine, which utilizes CMV-specific proteins (primarily glycoprotein B) to stimulate the immune system. Due to their limited components and non-replicating nature, these vaccines frequently need booster doses and adjuvants such as MF59 to enhance their immunogenicity. They were used in two phase 2 trials on healthy individuals [25,27] and in one phase 2 trial on transplant recipients [19]. This type of vaccine showed good antibody responses, though it produced a comparatively weaker T-cell response. Phase 1 and 2 trials on healthy individuals and a phase 2 trial on solid organ transplant recipients showed significantly higher levels of anti-gB and neutralizing antibodies in the vaccine group compared to the placebo group [19,24,26,27]. The infection rates per 100 person-years were significantly reduced by 50% in the vaccine group in the first phase 2 trial [25], but the same efficacy endpoint was not met in a subsequent phase 2 trial [27].

Additional vaccine types, which are not in an advanced experimental stage of development, include mRNA-based vaccines and live attenuated vaccines. Notably, mRNA vaccines are a novel, yet promising, approach, offering the major advantage of delivering multiple antigens within a single immunization and potentially encoding any antigen. A phase 1 study on healthy individuals showed a significant immunogenicity response in terms of neutralizing antibodies and the T-cell response [36].

Towne live attenuated vaccines were the first candidates investigated against CMV [11,12,13,14,15]. However, their development did not progress any further because they have shown only a moderate efficacy in transmission prevention and are considered unsafe for immunocompromised individuals due to the significant risk of viral reactivation. Two recent phase 1 trials evaluating the V160 live attenuated vaccine on healthy individuals reported significant humoral and T-cell responses [32,33].

Recent reviews have described the characteristics of different CMV vaccine platforms, focusing on different types of vaccine candidates under clinical development and emphasizing that developing a vaccine against CMV is considered a priority by the international scientific community [8,38,39,40,41]. Some reviews have focused on the prevention of congenital CMV [42] and on the prevention of CMV reactivation in transplant patients [43,44].

The strength of the present study is its description of the status of CMV vaccines under clinical development, considering not only the type of vaccine, but also the characteristics of the target population, differentiating between healthy individuals and transplant recipients. The reason for making this distinction is because the CMV-specific immune response to these vaccines is complex and varies depending not only on the type of vaccine, but notably on the immunological status of the individual. Therefore, to evaluate the vaccine endpoints in terms of safety, immunogenicity, and efficacy, it is essential to take these aspects into consideration. In fact, one main group prioritized for vaccination is high-risk immunocompromised individuals, particularly those undergoing an SOT or HSCT, in order to prevent CMV reactivation and reinfection after transplantation.

## 5. Conclusions

CMV seropositivity is widespread, but in healthy individuals, the virus does not usually cause disease. Two populations are primarily affected by CMV-associated morbidity and mortality: congenitally infected children and immunosuppressed individuals. For this reason, it represents a significant public health concern and the primary target populations for vaccination should be adolescents or women of childbearing age and immunocompromised individuals at high risk, such as recipients of an SOT or HSCT.

Adjuvanted gB vaccines as well as DNA vaccines targeting gB and pp65 have resulted in positive results in phase 2 clinical trials, while phase 3 was reached by the DNA vaccine platform.

In summary, although several CMV vaccine candidates have shown promise in terms of safety and specific immune responses, challenges remain in achieving a broad efficacy across diverse populations, particularly for immunocompromised patients. The current landscape is encouraging, but further research and development will be essential to realize an effective and widely applicable CMV vaccine in the near future.

## Figures and Tables

**Table 1:** a. General characteristics of included studies on transplant population. b. General characteristics of included studies on healthy population.

(**a**)								
**First Author** **Year** **Country**	**Type of Vaccine**	**N Study Population**	**Characteristics of the Population**	Study ID (NCT) * **Phase** **Blind Trial**	**N Experimental Arm**	**N Control Arm**	**Endpoints**	**Funding**
Plotkin 1984 USA [11]	Towne live attenuated	91	CMV+ and CMV− (31.9% CMV+) Renal transplant candidates	Double-blind	53	38	Safety Efficacy	National Institutes of Health and Hassel Foundation
Sachs 1984 USA [12]	Towne live attenuated	370	Age > 12y CMV+ and CMV− Renal transplant candidates	Double-blind	176	194	Immunogenicity	Merck, Sharp, and Dohme and the National Institutes of Health
Balfour 1985 USA [13]	Towne live attenuated	400	Age > 12y CMV+ and CMV− Renal transplant candidates	Double-blind	198	202	Safety Immunogenicity	National Institutes of Health
Plotkin 1991 USA [14]	Towne live attenuated	237	CMV+ and CMV− (39.2% CMV+) Renal transplant candidates	Double-blind	124	113	Safety Efficacy	National Institutes of Health and Food and Drug Administration
Plotkin 1994 USA UK [15]	Towne live attenuated	177	100% CMV− Renal transplant candidates	Double-blind	89	88	Immunogenicity Efficacy	Orphan Drug Program of the FDA
Kharfan-Dabaja 2012 USA [16]	TransVax DNA plasmid	108	Age: 25–63 (mean: 50) M: 48% 100% CMV+ Undergoing HSCT	NCT00285259 Phase 2 Double-blind	54	54	Safety Immunogenicity Efficacy	Vical and US National Institute of Allergy and Infectious Diseases
Vincenti 2018 USA [17]	ASP0113 DNA plasmid	149	Age ≥18 (mean: 49.3) M: 73% 100% CMV− Renal transplant recipients	NCT01974206 Phase 2 Double-blind	75	74	Safety Immunogenicity Efficacy	Astellas Pharma Global Development, Inc.
Ljungman 2021 USA [18]	ASP0113 DNA plasmid	514	Age ≥18 (range: 44–62) M: 57.9% 100% CMV+ HSCT recipients	NCT01877655 Phase 3 Double-blind	251	263	Safety Immunogenicity Efficacy	Astellas Pharma Global Development, Inc.
Griffiths 2011 UK [19]	gB/MF59 recombinant subunit	140	Age >18 M: 59% CMV+ and CMV− (50% CMV+) Renal or liver transplant candidates	NCT00299260 Phase 2 Double-blind	67	73	Safety Immunogenicity Efficacy	National Institute of Allergy and Infectious Diseases
Aldoss 2020 USA [20]	Triplex vector (poxvirus)	102	Age: 46–66 M: 63.7% 100% CMV+ HSCT recipients	NCT02506933 Phase 2 Double-blind	51	51	Safety Immunogenicity Efficacy	National Cancer Institute and Helocyte Inc.
Nakamura 2024 USA [21]	Pep vax (peptide-based)	61	Age: 18–75 M: 70.5% 100% CMV+ Undergoing HSCT	NCT02396134 Phase 2	32	29	Immunogenicity Efficacy	National Cancer Institute and Helocyte Inc.
(**b**)								
**First Author** **Year** **Country** **Ref**	**Type of Vaccine**	**N Study** **Population**	**Characteristics of the Population**	**Study ID (NCT)** **Phase** **Blind Trial**	**N Experimental Arm**	**N Control Arm**	**Endpoints**	**Funding**
Pass 1999 USA [22]	gB/MF59 gB/Alum recombinant subunit	46	Age: 21–50 M: 37% 100% CMV−	Phase 1 Double-blind	40 30 gB/MF59 10 gB/Alum	6	Safety Immunogenicity	Chiron Vaccines
Drulak 2000 USA [23]	CHIRON gB/F59 recombinant subunit	114	100% CMV+	Group 1 blind	97	14	Safety Immunogenicity	Not reported
Bernstein 2002 USA [24]	gB/MF59 recombinant subunit ALVAC-gB vector (canarypox virus)	105	Age: 18–45 100% CMV−		90 32 gB/MF59 32 ALVAC-gB, gB/MF59 26 gB/MF59 + ALVAC-CMVgB	15	Safety Immunogenicity	National Institutes of Health
Pass 2009 USA [25]	gB/MF59 recombinant subunit	464	Age: 14–40 (median: 20) F: 100% 100% CMV−	NCT00125502 Phase 2 Double-blind	234	230	Safety Efficacy	Sanofi Pasteur, University of Alabama at Birmingham grant from the National Institute of Allergy and Infectious Diseases and from the National Center for Research Resources
Sabbaj 2011 USA [26]	gB/MF59 recombinant subunit	150	Age: 14–40 (median: 26) F: 100% 100% CMV+	Phase 1 Double-blind	120	30	Immunogenicity	Sanofi Pasteur and Public Health Service grant from the National Institutes of Health National Center for Research Resources
Bernstein 2016 USA [27]	gB/MF59 recombinant subunit	409	Age: 12–18 (median: 15) F: 100% 100% CMV−	NCT00133497 Phase 2 Double-blind	195	207	Safety Immunogenicity Efficacy	Federal funds from the NIAID/NIH/HHS and from the Biomedical Advanced Research and Development Authority, Department of Health and Human Services
Berencsi 2001 USA [28]	ALVAC-pp65 vector (canarypox virus)	27	Age: 18–35 M: 18.5% CMV+ and CMV− (15% CMV+)	Phase 1	14	9	Safety Immunogenicity	Aventis Pasteur
Bernstein 2009 USA [29]	Alphavirus replicon vector	40	Age: 18–45 M: 37.5% 100% CMV−	NCT00439803 Phase 1 Double-blind	32	8	Safety Immunogenicity	AlphaVax
Schwendinger 2020 USA [30]	HB-101 vector (LCMV)	54	Age: 18–45 (30.4 ± 7.5) 4:5 male/female ratio 100% CMV−	NCT02798692 Phase 1 Double-blind	42	12	Safety Immunogenicity	Hookipa Pharma, Inc. Austrian Research Promotion Agency
Heineman 2006 USA [31]	Towne/Toledo chimeric live attenuated	25	Heathy volunteers Age: 18–60 100% CMV+ M: 68%	Phase 1 Double-blind	20	5	Safety Immunogenicity Efficacy	MedImmune Vaccines National Institute of Allergy and Infectious Diseases
Adler 2019 USA [32]	V160 live attenuated	190	Age > 18, mean: 44.1 (±14.6) M: 42.6% CMV+ and CMV− (50% CMV+)	NCT01986010 Phase 1 Double-blind	145	43	Safety Immunogenicity	Merck Sharp & Dohme Corp
Murata 2023 Japan [33]	V160 live attenuated	18	Age: 20–64 (mean 36.4) M: 100% CMV+ and CMV− (50% CMV+)	NCT03840174 Phase 1 Double-blind	12	6	Safety Immunogenicity	Merck Sharp & Dohme LLC,
Bonate 2020 USA [34]	ASP0113 DNA plasmid	44	Mean age: 40.7 ± 12.0 M: 72.7% CMV+ and CMV− (33% CMV+)	NCT02103426 Phase 1b Subject-blind	37	7	Safety Immunogenicity	Astellas Pharma Global Development, Inc.
Langley 2024 Canada [35]	VBI-1501/Alum VBI-1501 virus-like particles	128	Age: 18–40 M: 41% 100% CMV−	NCT02826798 Phase 1 Double-blind	77 VBI-1501/Alum 25 VBI-1501	26	Safety Immunogenicity	VBI Vaccines
Fierro 2024 USA [36]	m-RNA-1647	154	Age: 18–49 M: 36.6% CMV+ and CMV− (48% CMV+)	NCT03382405 Phase 1 Double-blind	118	36	Safety Immunogenicity	Moderna

* NCT: national clinical trial number; gB: glycoprotein B; HSCT: hematopoietic stem cell transplantation; LCMV: lymphocytic choriomeningitis virus; CMV+: CMV-seropositive; CMV−: CMV-seronegative.

## Supplementary Materials

The following supporting information can be downloaded at: https://www.mdpi.com/article/10.3390/vaccines13010085/s1, Table S1. Search strategy. Table S2. PRISMA Checklist (2020). Table S3. Endpoint assessment.
