# Supplementary material for: Safety, Immunogenicity, and Efficacy of Cytomegalovirus Vaccines: A Systematic Review of Randomized Controlled Trials
Source: Vaccines (Basel). 2025 Jan 17;13(1):85. doi: 10.3390/vaccines13010085 (PMC11768780; doi:10.3390/vaccines13010085)
Supplement: Supplementary file 1 [file vaccines-13-00085-s001.zip › Supplementary Table S1. Search strategy.pdf]

Supplementary Table S1. Search strategy

Database	Search details
**PubMed**	((CMV vaccin*) OR (Cytomegalovirus vaccin*)) AND (efficacy [title/abstract] OR immunogenicity [title/abstract])
**Web of Science**	Topic: (CMV vaccin*) OR (Cytomegalovirus vaccin*)<br>AND<br>Topic: efficacy OR immunogenicity<br>AND<br>Topic: Randomized Controlled Trial
**Scopus**	TAK: cmv AND vaccine* OR cytomegalovirus AND vaccine*<br>AND<br>TAK: efficacy OR immunogenicity<br>AND<br>TAK: randomized AND controlled AND trial
**Cochrane**	((CMV vaccin*) OR (Cytomegalovirus vaccin*)) AND (efficacy OR immunogenicity) in Title Abstract Keyword - (Word variations have been searched)
