# Supplementary material for: Safety, Immunogenicity, and Efficacy of Cytomegalovirus Vaccines: A Systematic Review of Randomized Controlled Trials
Source: Vaccines (Basel). 2025 Jan 17;13(1):85. doi: 10.3390/vaccines13010085 (PMC11768780; doi:10.3390/vaccines13010085)
Supplement: Supplementary file 1 [file vaccines-13-00085-s001.zip › Supplementary Table S3. Endpoint assessment.pdf]

Supplementary Table S3. Endpoint assessment.

	**Transplant population**	**Healthy population**
**Safety**	[11,13,14,16-20]	[22-25,27-36]
**Immunogenicity**	[12,13,15-21]	[22-24,26-36]
**Efficacy**	[11,14-21]	[25,27,31]
